# Supplementary material for: A cofactor-induced repressive type of transcription factor condensation can be induced by synthetic peptides to suppress tumorigenesis
Source: EMBO J. 2024 Oct 2;43(22):5586–612. doi: 10.1038/s44318-024-00257-4 (PMC11574045; doi:10.1038/s44318-024-00257-4)
Supplement: Supplementary file 1 — Appendix [file 44318_2024_257_MOESM1_ESM.pdf]

Appendix

Contents	Pages
Appendix Figure S1	2-3
Appendix Figure S2	4-5
Appendix Figure S3	6
Appendix Figure S4	7-8
Appendix Figure S5	9-10
Appendix Figure S6	11-12
Appendix Figure S7	13
Appendix Figure S8	14
Appendix Table S1	15

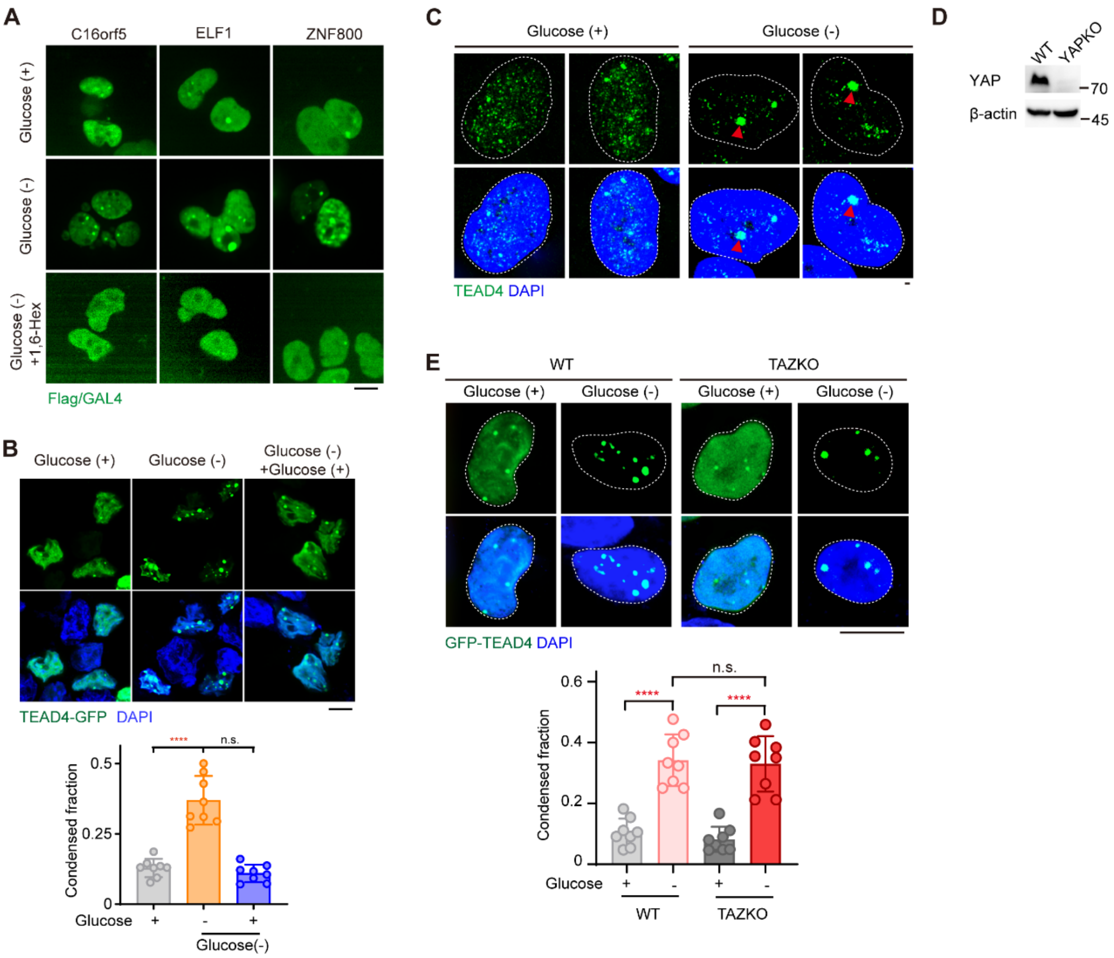

Appendix Figure S1. Glucose Deprivation Induces Formation of TF Condensates

A. Representative images of TF condensates in HEK293FT cells transfected with the indicated Flag- or Gal4-tagged plasmids. Scale bar, 10 μm. **B.** Fluorescence images of GFP-TEAD4 condensates in glucose starvation-treated HEK293FT cells with or without glucose supplement (upper), and quantification of TEAD4 condensed fraction (lower). The data were analyzed using one-way ANOVA, followed by the Tukey's post-hoc test. n.s., no significance; ****, $p < 0.0001$. Scale bar, 10 μm. **C.** Fluorescence images of GFP-TEAD4 condensates in HGC-27 cells with or without their being subjected to glucose starvation. Scale bar, 1 μm. **D.** Immunoblotting showing YAP protein levels in wild-type (WT) and YAPKO cells. **E.** Fluorescence images of GFP-TEAD4 condensates in wild-type (WT) and TAZ-knockout (TAZKO) cells with or without their being subjected to glucose deprivation for 12 h (upper), and quantification of TEAD4 condensed fraction (bottom). The data were analyzed using one-way ANOVA,

followed by the Tukey's post-hoc test. n.s., no significance; ****, $p < 0.0001$. Scale bar, 10 μm.

Related to **Figure 1.**

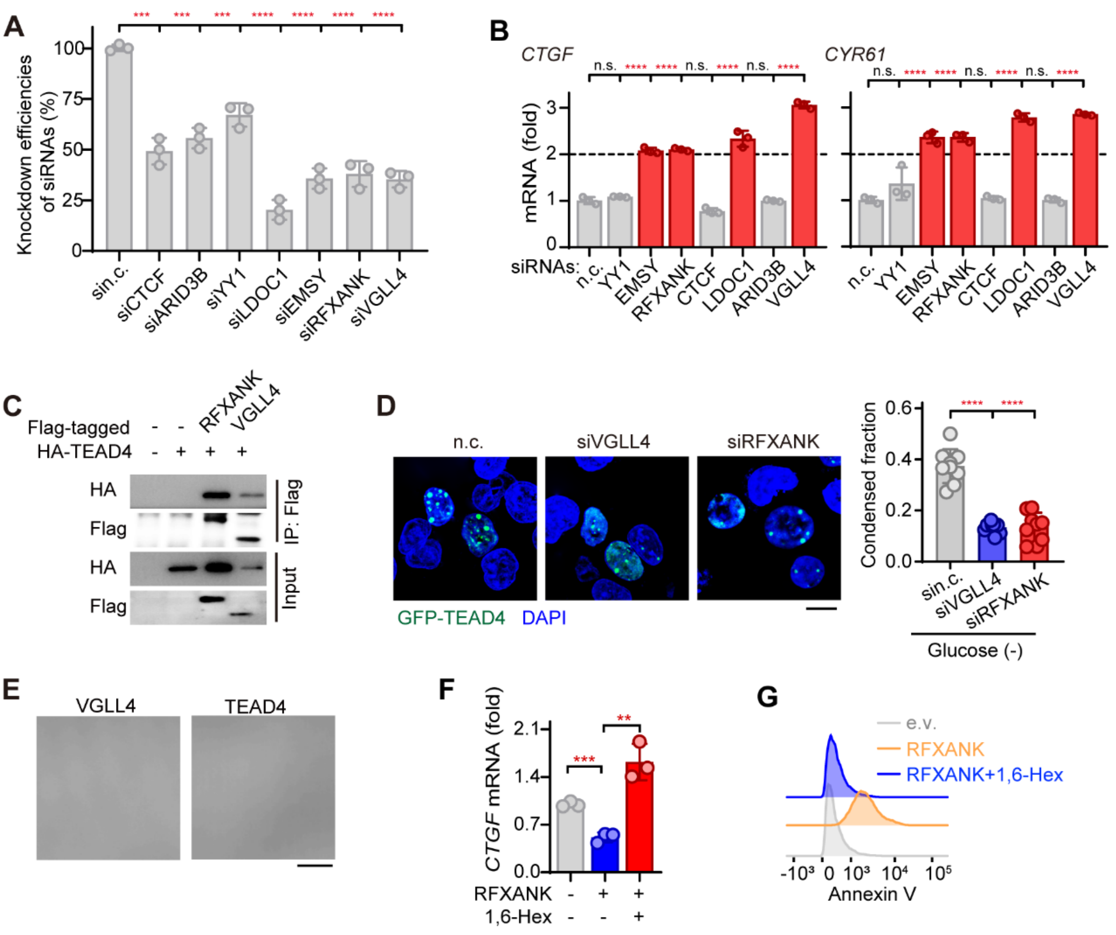

Appendix Figure S2. VGLL4 and RFXANK as TEAD4 Phase Separation Inducers

A. Realtime-PCR (RT-PCR) analysis of the seven indicated siRNAs knockdown efficiencies in HGC-27 cells using *ACTB* as an internal control (n = 3/group). The data were analyzed using one-way ANOVA, followed by the Tukey's post-hoc test. ***, p < 0.001; ****, p < 0.0001. **B.** mRNA levels of *CTGF* and *CYR61* in HGC-27 cells transfected with the indicated siRNAs (n = 3/group). The cutoff value was >2 fold in mRNA change. The data were analyzed using one-way ANOVA, followed by the Tukey's post-hoc test. n.s., no significance; ****, p < 0.0001. **C.** Co-immunoprecipitation (co-IP) analysis of the interaction between TEAD4 and RFXANK or VGLL4 in HEK293FT cells. **D.** Fluorescence images of GFP-TEAD4 condensates in VGLL4- or RFXANK-knockdown cells upon their being subjected to glucose starvation (left), and quantification of TEAD4 condensed fraction (right). The data were analyzed using one-way ANOVA, followed by the Tukey's post-hoc test. ****, p < 0.0001. Scale bar, 10 μm. **E.** Droplet formation of indicated purified proteins. Scale bar,

10 μm. **F.** Transcription of *CTGF* in RFXANK-overexpressing HEK293FT cells treated with or without 1,6-Hex ($n = 3$/group). Data are presented as means ± SD. Significance was tested using one-way ANOVA, followed by the Tukey's post-hoc test. **$p < 0.01$, ***$p < 0.001$. **G.** Annexin V staining of RFXANK-overexpressing HEK293FT cells treated with or without 1,6-Hex. e.v., empty vector.

Related to **Figure 2.**

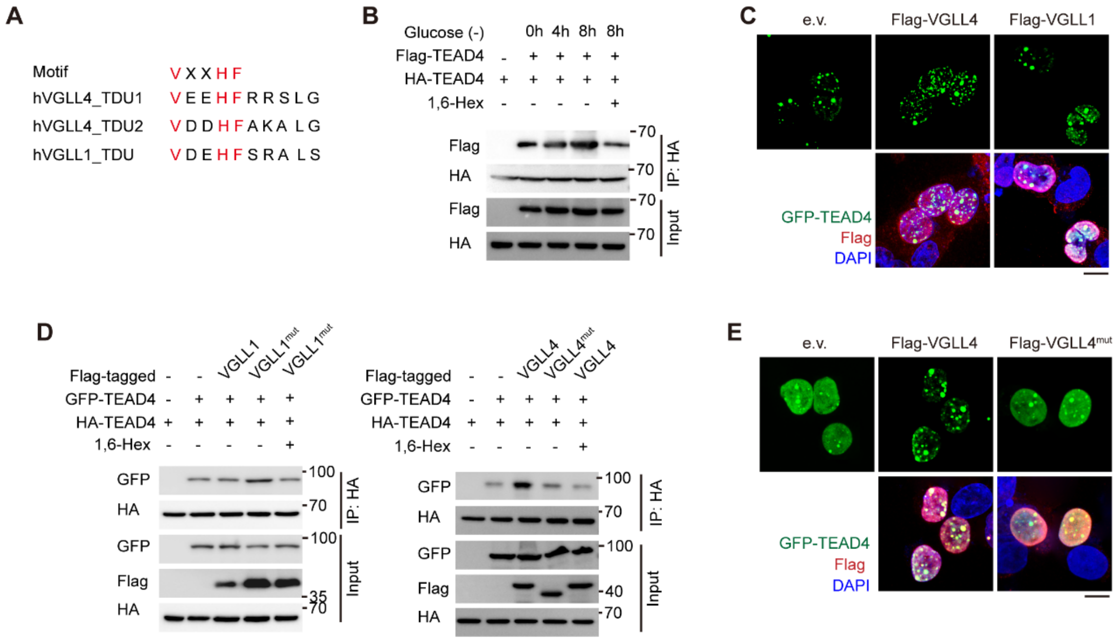

Appendix Figure S3. Glucose Deprivation Promotes TEAD4 Oligomerization

A. Sequence alignment of TDU domains from VGLL1 and VGLL4. Key amino acid residues for binding to TEAD4 are shown in red. **B.** Results of Co-IP assay testing for TEAD4 oligomerization, specifically for the binding of HA-TEAD4 to Flag-TEAD4 in cells subjected to glucose limitation at indicated time points and with or without 1,6-Hex treatment. **C.** Fluorescence images of TEAD4 condensates in VGLL1- or VGLL4-overexpressing HEK293FT cells. Scale bar, 10 μm. **D.** Co-IP analysis of TEAD4 oligomerization in HEK293FT cells transfected with VGLL1, VGLL4 or their mutants and with or without 1,6-Hex treatment. VGLL1^{mut} is a construct in which TDU1 (amino acid residues 206–230) of VGLL4 was added to the N-terminal of wildtype VGLL1 to create a VGLL1 version with two TDUs. VGLL4^{mut} is a construct in which TDU1 (amino acid residues 206–229) of VGLL4 was deleted to create a VGLL4 version with one TDU. **E.** Fluorescence images of TEAD4 condensates in HEK293FT cells transfected with VGLL4 or its mutant. Scale bar, 10 μm.

Related to **Figure 3.**

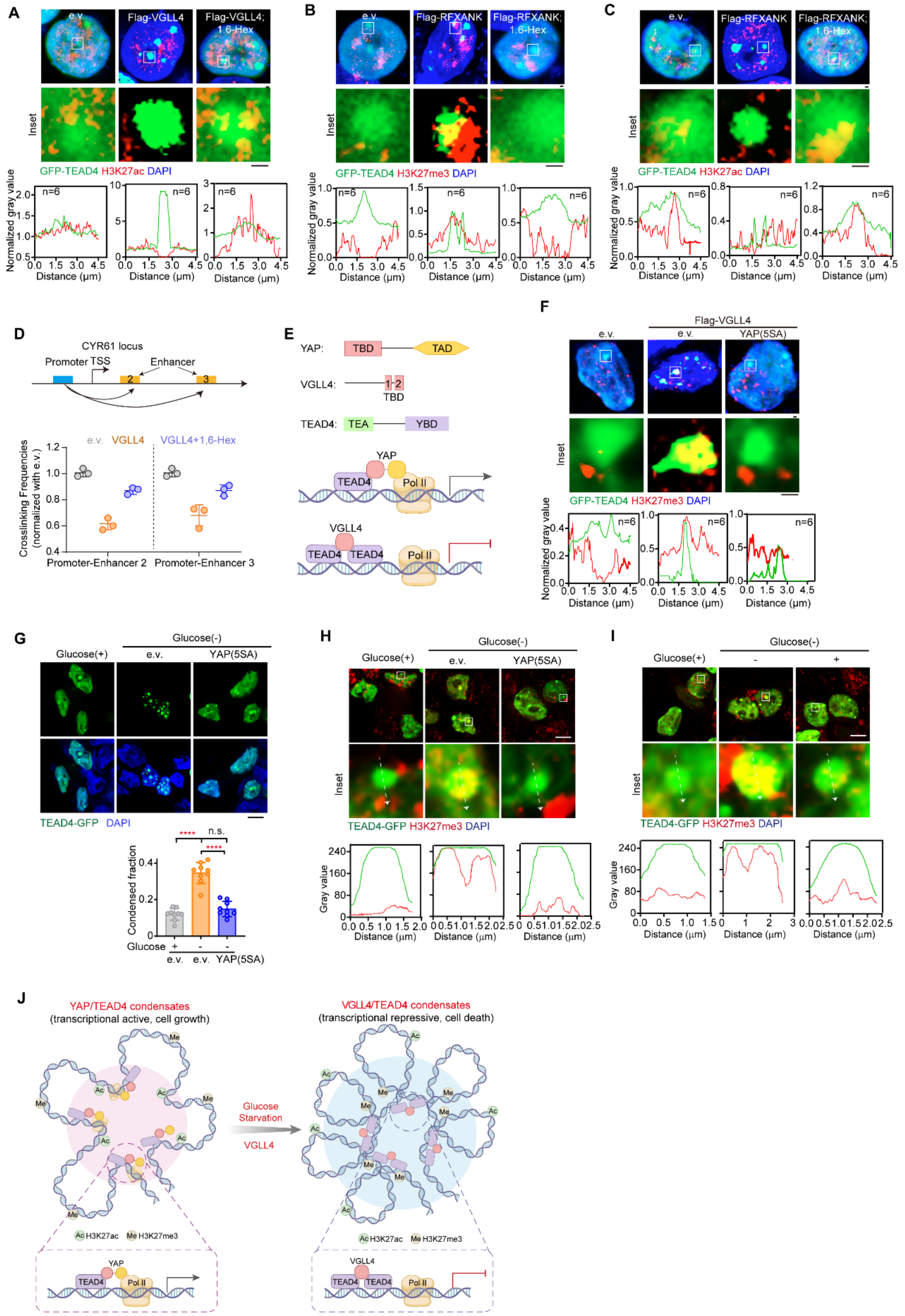

Appendix Figure S4. TEAD4 Condensates Are Correlated with Transcriptional Repression

A. Representative fluorescence images of GFP-TEAD4 and H3K27ac in VGLL4-overexpressing HEK293FT cells treated with or without 1,6-Hex (upper panel), and quantification of the fluorescence intensity levels of 6 cells with indicated color scheme (lower panel). Scale bar, 1 μm. **B.** Representative images of GFP-TEAD4 and H3K27me3 in RFXANK-overexpressing HEK293FT cells treated with or without 1,6-Hex (upper panel), and quantification of the fluorescence intensity levels of 6 cells with indicated color scheme (lower panel). Scale bar, 1 μm. **C.** Confocal microscopy images of GFP-TEAD4 and H3K27ac in RFXANK-transfected HEK293FT cells treated with or without 1,6-Hex(upper panel) and quantification of fluorescence intensity levels of 6 cells with indicated color scheme (lower panel). Scale bar, 1 μm. **D.** 3C analysis to assess the distance of *CYR61* in VGLL4-overexpressing HEK293FT cells treated with or without 1,6-Hex. **E.** Domain architecture and binding models of YAP, VGLL4 and TEAD4. **F.** Immunofluorescence staining (upper and middle, representative images with zoom-in; lower, quantification of fluorescence intensity levels of 6 cells with indicated color scheme) of GFP-TEAD4 and H3K27me3 in VGLL4-expressed HEK293FT cells with or without YAP (5SA) co-transfection. Scale bar, 1 μm. **G.** Fluorescence images of GFP-TEAD4 condensates in glucose starvation-treated HEK293FT cells with or without YAP(5SA) expression (top), and quantification of TEAD4 condensed fraction (bottom). The data were analyzed using one-way ANOVA, followed by the Tukey's post-hoc test. n.s., no significance; ****, $p < 0.0001$. Scale bar, 10 μm. **H.** Fluorescence images of GFP-TEAD4 and H3K27me3 in glucose-deprived HEK293FT cells transfected with or without YAP(5SA). Scale bar, 10 μm. **I.** Fluorescence images of GFP-TEAD4 and H3K27me3 in glucose-deprived HEK293FT cells with or without glucose re-supplement. Scale bar, 10 μm. **J,** Cartoon presentation of the two forms (transcriptionally active or repressive) of TEAD4 LLPS driven by YAP or VGLL4.

Related to **Figure 4.**

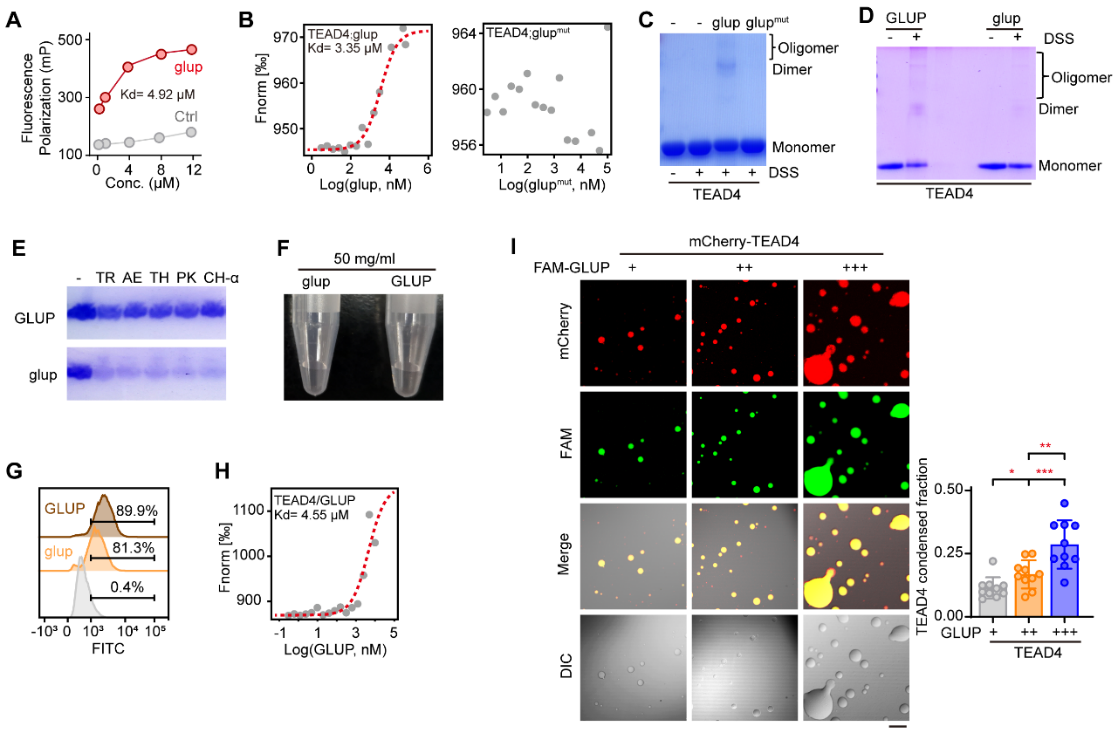

Appendix Figure S5. Optimization of the Linker Peptide

A. Fluorescence polarization (FP) assay to detect the interaction of purified TEAD4 protein with FAM-labeled glup. mP, millipolarization (FP values). **B.** Microscale thermophoresis (MST) assay binding curves for the interaction of purified TEAD4 protein with glup or glup^{mut}. Kd values are shown. glup^{mut}, an interaction-dead control peptide with the sequence RRVCVAAAASLSLR. **C.** Crosslinking assay of TEAD4 oligomerization in the presence of glup or glup^{mut} after DSS treatment. **D.** Crosslinking assay of TEAD4 oligomerization in the presence of glup or GLUP with or without DSS treatment. **E.** Gels assessing protein stability levels of glup and GLUP after they were treated with indicated proteases. TR, trypsin; AE, actinase E; TH, thermolysin; PK, proteinase K; CH-α, chymotrypsin-α. **F.** Photograph of vials of GLUP and glup peptides in ddH_2O, for assessing their solubility levels. **G.** Flow cytometry analysis of cell permeability of FITC-labeled glup or GLUP in HEK293FT cells. **H.** MST assay showing the interaction of GLUP with TEAD4. **I.** Droplet formation of mCherry-TEAD4 with increasing doses of FAM-GLUP (left) and quantification of TEAD4 condensed fraction (right). GLUP (+), 20 μM; GLUP (++), 40 μM; GLUP (+++), 80 μM. The data were analyzed using one-way ANOVA, followed by the Tukey's post-hoc test. *, $p < 0.05$; **,

$p < 0.01$; ***, $p < 0.001$. Scale bar, 10 μm.

Related to **Figure 5.**

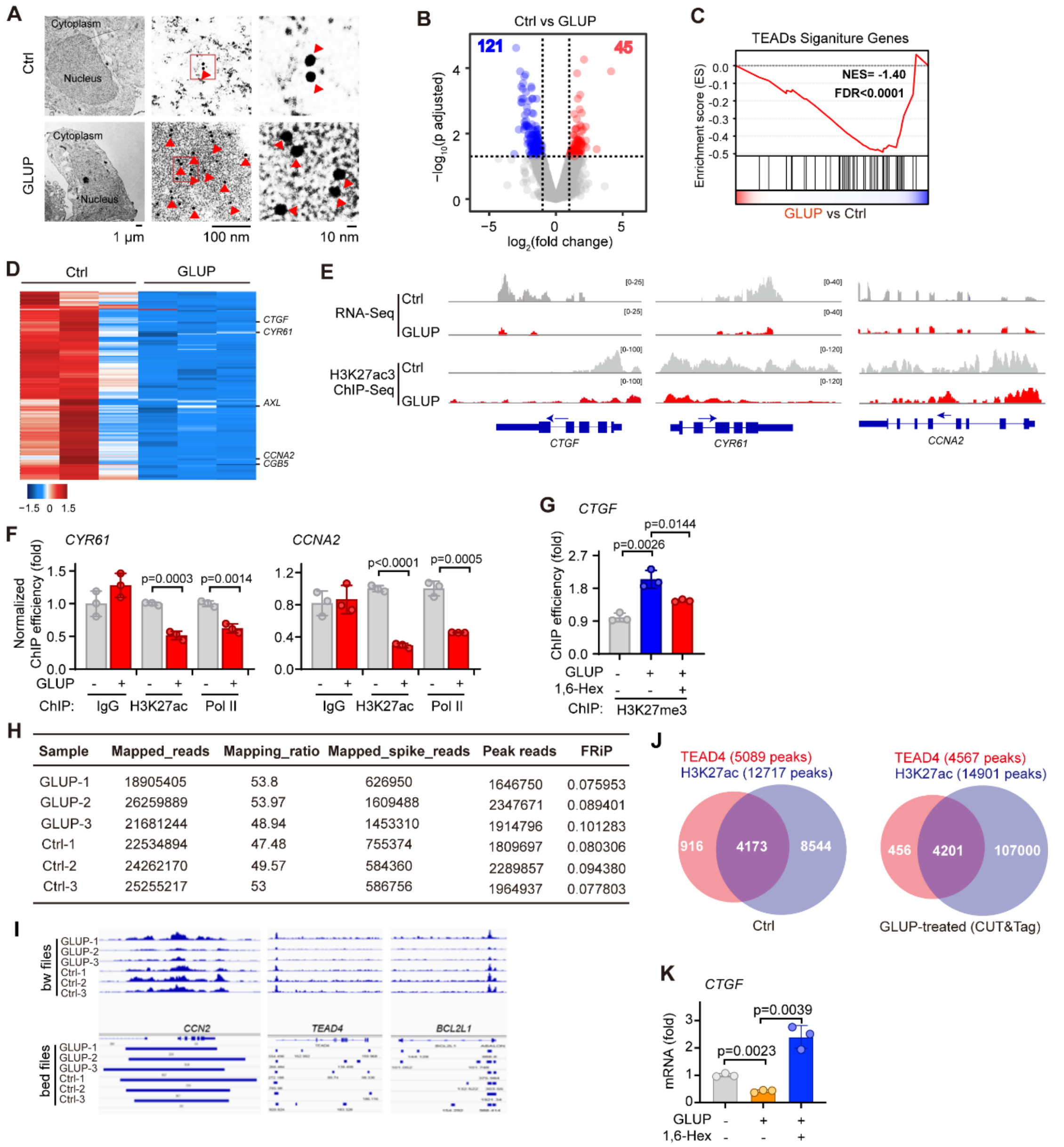

Sample	Mapped_reads	Mapping_ratio	Mapped_spike_reads	Peak reads	FRiP
GLUP-1	18905405	53.8	626950	1646750	0.075953
GLUP-2	26259889	53.97	1609488	2347671	0.089401
GLUP-3	21681244	48.94	1453310	1914796	0.101283
Ctrl-1	22534894	47.48	755374	1809697	0.080306
Ctrl-2	24262170	49.57	584360	2289857	0.094380
Ctrl-3	25255217	53	586756	1964937	0.077803

Appendix Figure S6. Functional Assessment of GLUP

A. Colloidal gold IEM images depicting TEAD4 particles in GLUP-treated HGC-27 cells. The red arrows denote TEAD4 particles stained by colloidal-gold-conjugated primary antibody. Scale bars: 1 μm for the left images, 100 nm for the middle ones, and 10 nm for the right ones. **B.** Volcano plot of altered genes in HGC-27 cells treated with GLUP (n = 3/group). Red dots represent up-regulated genes while blue ones represent down-regulated genes. **C.** Gene set enrichment analysis (GSEA) of TEAD signature genes in GLUP-treated HGC-27 cells. Normalized enrichment score (NES) and FDR are shown. **D.** Heatmap for down-regulated genes in control or GLUP-treated HGC-27

cells (n = 3). **E.** Integrative Genomics Viewer (IGV) snapshot depicting RNA-Seq and ChIP-Seq signals of the indicated genes in GLUP-treated cells. Signals are plotted on a normalized read per million (RPM) bases. **F.** Enrichment of H3K27ac3 and polymerase II (Pol II) on the promoters of *CYR61* and *CCNA2* in GLUP-treated cells as determined by performing chromatin immunoprecipitation-quantitative PCR (ChIP-qPCR) (n = 3/group). Data are presented as means ± SD. Significance was tested using one-way ANOVA, followed by the Tukey's post-hoc test. **p < 0.01, ***p < 0.001. ****p < 0.0001. **G.** ChIP-qPCR-measured enrichment of H3K27me3 on *CTGF* promoter in GLUP-treated cells after they were treated with 1,6-Hex (n = 3/group). Data are presented as means ± SD. Significance was tested using one-way ANOVA, followed by the Tukey's post-hoc test. *p < 0.05, **p < 0.01. **H.** FRiP values for TEAD4 ChIP-Seq assay using CUT&Tag strategy for triplicates with control or GLUP treatment. **I.** IGV showing the single peak for *CTGF*, *TEAD4* and *BCL2L1* in TEAD4 ChIP-Seq assay using CUT&Tag strategy for triplicates with control or GLUP treatment. **J.** Analysis of the binding of H3K27ac to the TEAD4-specific motifs upon GLUP treatment. **K.** mRNA levels of *CTGF* in GLUP-treated cells with or without 1,6-Hex (n = 3/group). Data are presented as means ± SD. Significance was tested using one-way ANOVA, followed by the Tukey's post-hoc test. **p < 0.01.

Related to **Figure 6.**

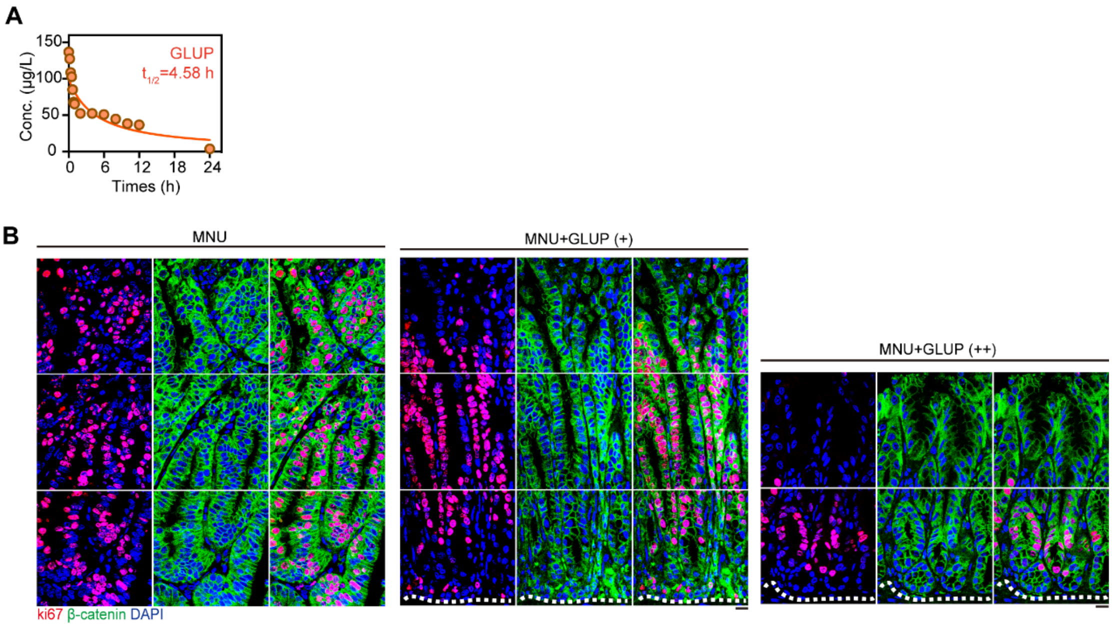

Appendix Figure S7. Evaluation of GLUP *in vivo*

A. Half-lives ($t_{1/2}$) of GLUP in plasma of mice. GLUP: 10 mg/kg per mice. **B.** Immunofluorescence images of β-catenin and Ki67 in MNU-generated GC tumors from GLUP-treated mice. Scale bar, 10 μm.

Related to **Figure 7.**

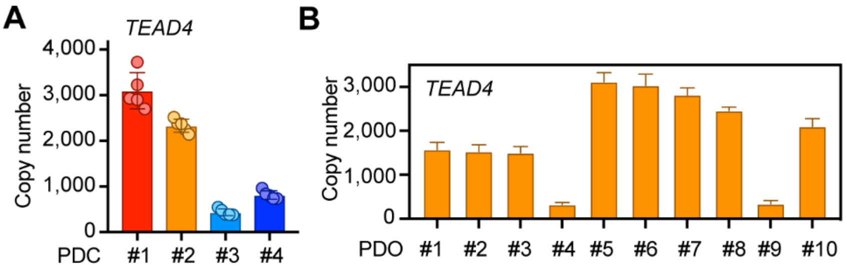

C

Inde	Saline (10 ml/kg)	GLUP (1 mg/kg)	GLUP (10 mg/kg)
White blood cell (10^9/L)	6.9±1.1	6.0±1.2	4.7±0.9*
Ret blood cell (10^{12}/L)	11.0±0.3	9.3±0.7**	9.4±0.3***
Hemoglobin	169.0±5.9	142.5±8.9**	141.0±5.1***
Platelet (10^9/L)	311.3±24.1	946.5±147.3**	997.5±101.7**
Lymphocytes (10^9/L)	6.5±1.0	4.9±1.4	3.8±0.7**
Monocytes (10^9/L)	0.1±0.1	0.2±0.1**	0.2±0.4**
Neutrophil (10^9/L)	0.21±0.1	0.5±0.2*	0.6±0.2***
Albumin (g/L)	23.1±4.41	24.1±2.1	23.8±1.9
Glucose (mmol/L)	8.71±0.9	11.9±0.7**	11.2±1.1**
Creatinnine (umol/L)	17.1±2.5	15.4±1.4	14.2±1.6
Urea (mmol/L)	8.0±0.8	7.3±1.0	7.5±1.0
AST (U/L)	97.0±3.5	82.3±10.1	76.5±12.9*
ALT (U/L)	34.5±1.7	36.0±7.4	30.0±3.6

Appendix Figure S8. Evaluation of the GLUP Therapy

A. Copy numbers of *TEAD4* transcripts in 4 PDCs. Data are presented as means ± SD. **B.** Copy numbers of *TEAD4* transcripts in 10 PDOs. Data are presented as means ± SD. **C.** Biochemical analysis of blood from mice treated with indicated doses of GLUP. Data are presented as means ± SD. *$p < 0.05$, **$p < 0.01$, ***$p < 0.001$.

Related to **Figure 8.**

Appendix Table S1. The Sequence of Indicated Primers

Gene name (human)	Forward (5'-3')	Reverse (5'-3')
CTGF	AAAAGTGCATCCGTACTCCCA	CCGTCGGTACATACTCCACAG
CYR61	GGTCAAAGTTACCGGGCAGT	GGAGGCATCGAATCCCAGC
CTCF	GAAGCCTCCAAAGCCAAC	GCACTTGTGTGGTCTCTCATC
ARID3B	GATGCCAGAGAGAAGCAG	GTCTCCCAGCTGTGGC
YY1	CAGATTCTCATCCCGGTG	CCGCTGAGGTAACTCTTCTTG
LDOC1	CTCATGGAACAGCTGCG	CATGGCGTCGTTGCAG
EMSY	CTGGAGCTGCAACCTATG	CAATCACGTTGGGCTTG
RFXANK	CTGCCTCAGAACTTGGG	CATCCGGTTCAGGATTC
VGLL4	AACTGCAACCTCTCGCACTG	GAGTGGGTGTCGCTGTTGAA
ACTB	ATCATGAAGTGTGACGTGGA	CTCAGGAGGAGCAATGATCT
3C		
CYR61 locus:		
Anchor primer: 5’-TGGGGTTCTACAGTCGTAAAAG-3’	Enhancer 2 primer: 5’-AAAGAGAGCAGAGATGAGAAACAC-3’	Enhancer 3 primer: 5’-GGGAGATGCCTTTGCTTTG-3’
MYC locus:		
Anchor primer: 5’-CGGTAATGGCAAACGTGAA-3’	Enhancer 1 primer: 5’-GGGGAGTACATTAGAGGAACAAA-3’	Enhancer 4 primer: 5’-GTCCTATCAGCCAGAACTTAGCC-3’
