# Supplementary material for: A cofactor-induced repressive type of transcription factor condensation can be induced by synthetic peptides to suppress tumorigenesis
Source: EMBO J. 2024 Oct 2;43(22):5586–612. doi: 10.1038/s44318-024-00257-4 (PMC11574045; doi:10.1038/s44318-024-00257-4)
Supplement: Supplementary file 7 — Movie EV2 [file 44318_2024_257_MOESM7_ESM.zip › Movie EV2.docx]

**Movie EV2.** Live-cell movie of HGC-27 cells treated with FAM (a fluorescent moiety)-labeled GLUP merged with nuclear region stained with DAPI for 30s. FAM-GLUP: 10 μg/ml (5 μM).
