# Supplementary figures and images for: A cofactor-induced repressive type of transcription factor condensation can be induced by synthetic peptides to suppress tumorigenesis
Source: EMBO J. 2024 Oct 2;43(22):5586–612. doi: 10.1038/s44318-024-00257-4 (PMC11574045; doi:10.1038/s44318-024-00257-4)

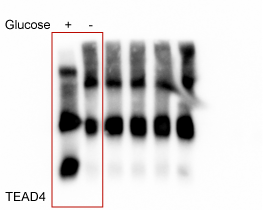

Supplement: Supplementary file 10 — Source data Fig. 3 [file 44318_2024_257_MOESM10_ESM.zip › Figure 3/3C/Native gel_endogenous TEAD4.tif]

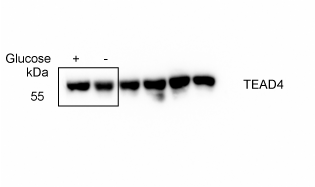

Supplement: Supplementary file 10 — Source data Fig. 3 [file 44318_2024_257_MOESM10_ESM.zip › Figure 3/3C/SDS_endogenous TEAD4.tif]

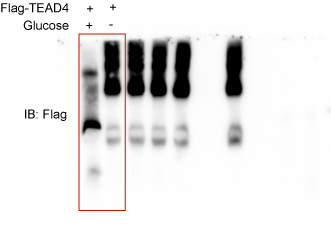

Supplement: Supplementary file 10 — Source data Fig. 3 [file 44318_2024_257_MOESM10_ESM.zip › Figure 3/3C/Native gel_exogenous Flag-TEAD4.tif]

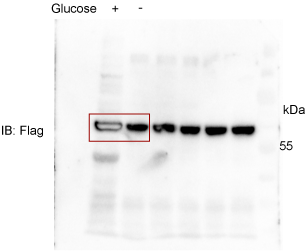

Supplement: Supplementary file 10 — Source data Fig. 3 [file 44318_2024_257_MOESM10_ESM.zip › Figure 3/3C/SDS_enxogenous Flag-TEAD4.tif]

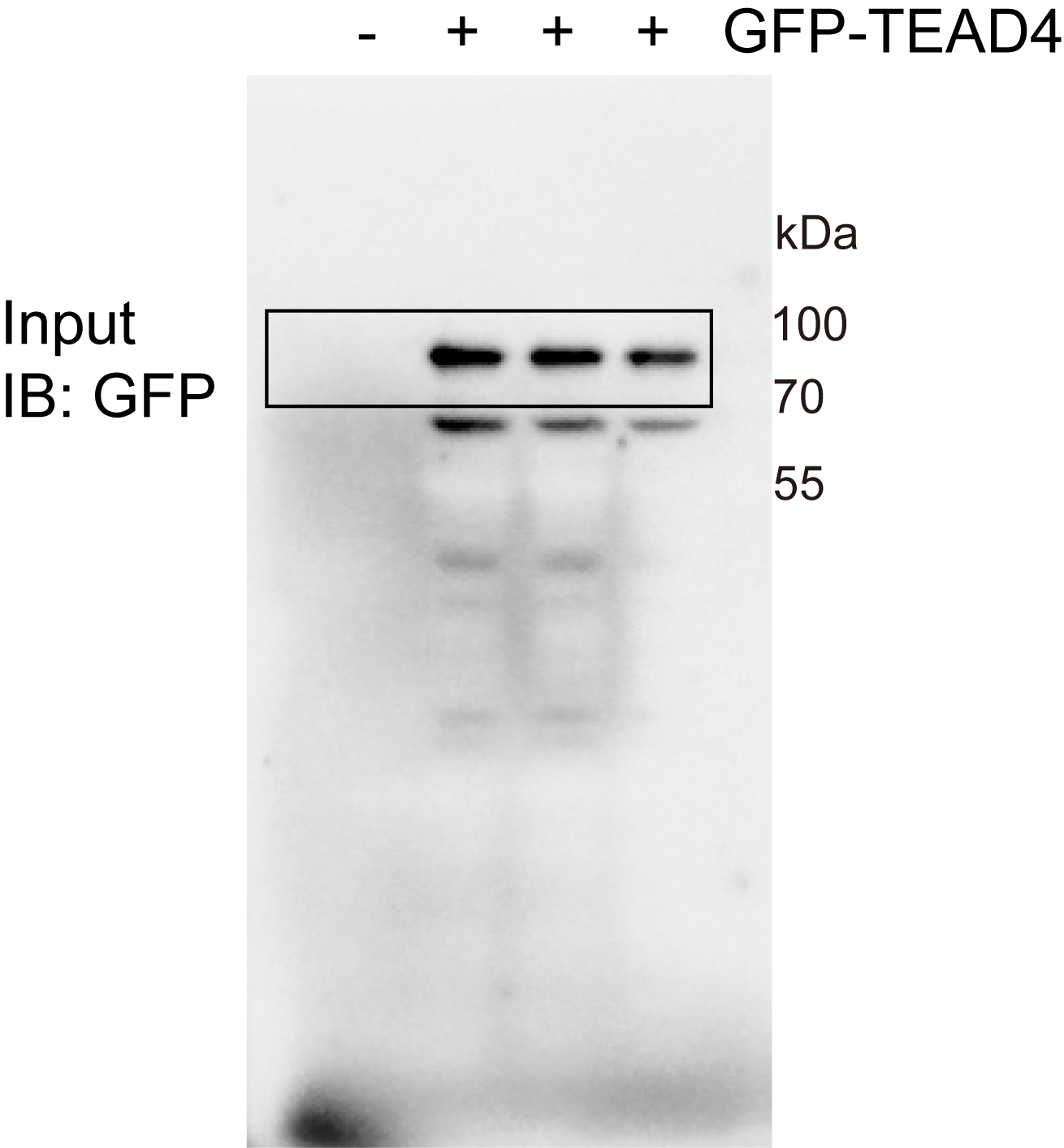

Supplement: Supplementary file 10 — Source data Fig. 3 [file 44318_2024_257_MOESM10_ESM.zip › Figure 3/3D/Input_IB_GFP.tif]

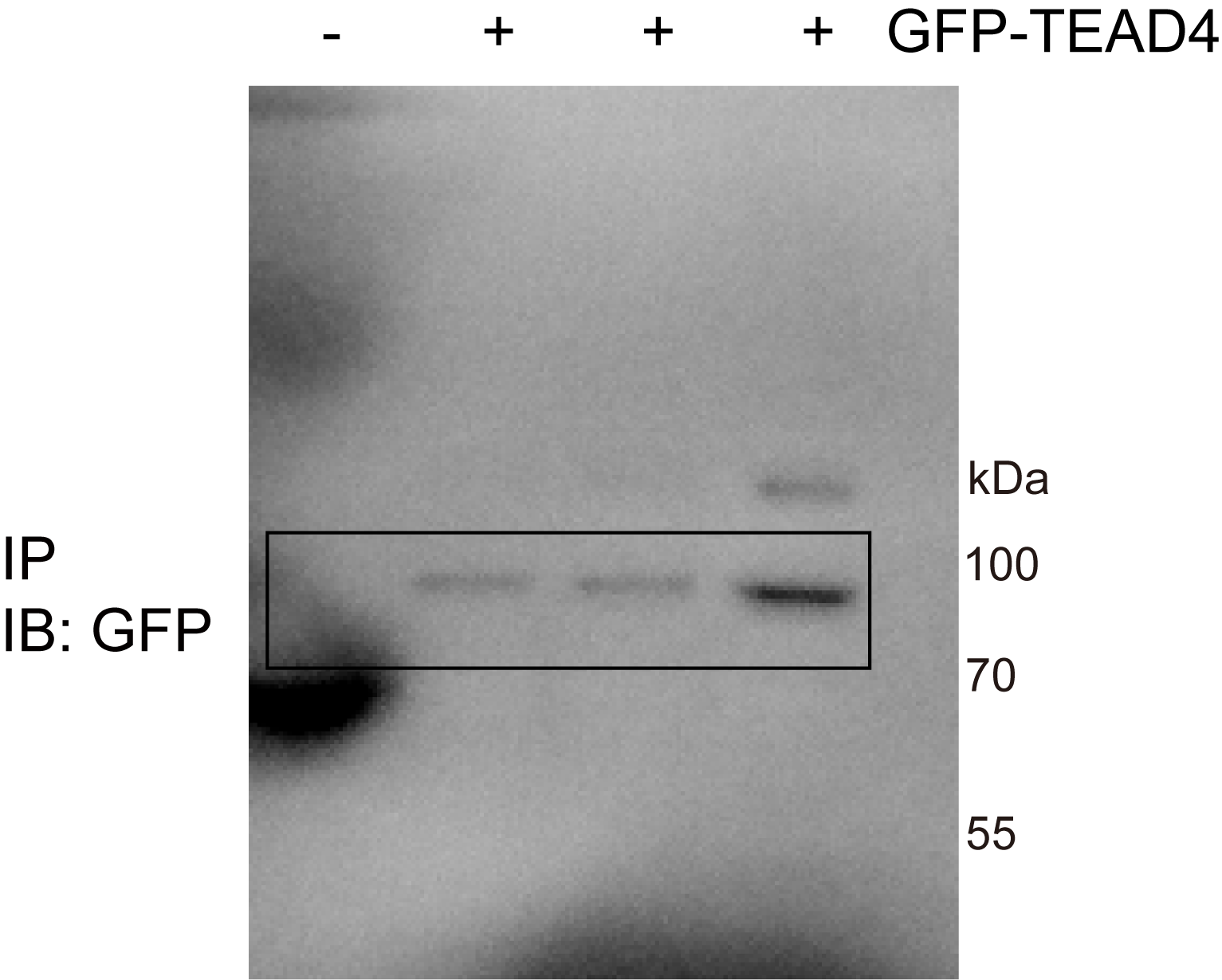

Supplement: Supplementary file 10 — Source data Fig. 3 [file 44318_2024_257_MOESM10_ESM.zip › Figure 3/3D/IP_IB_GFP.tif]

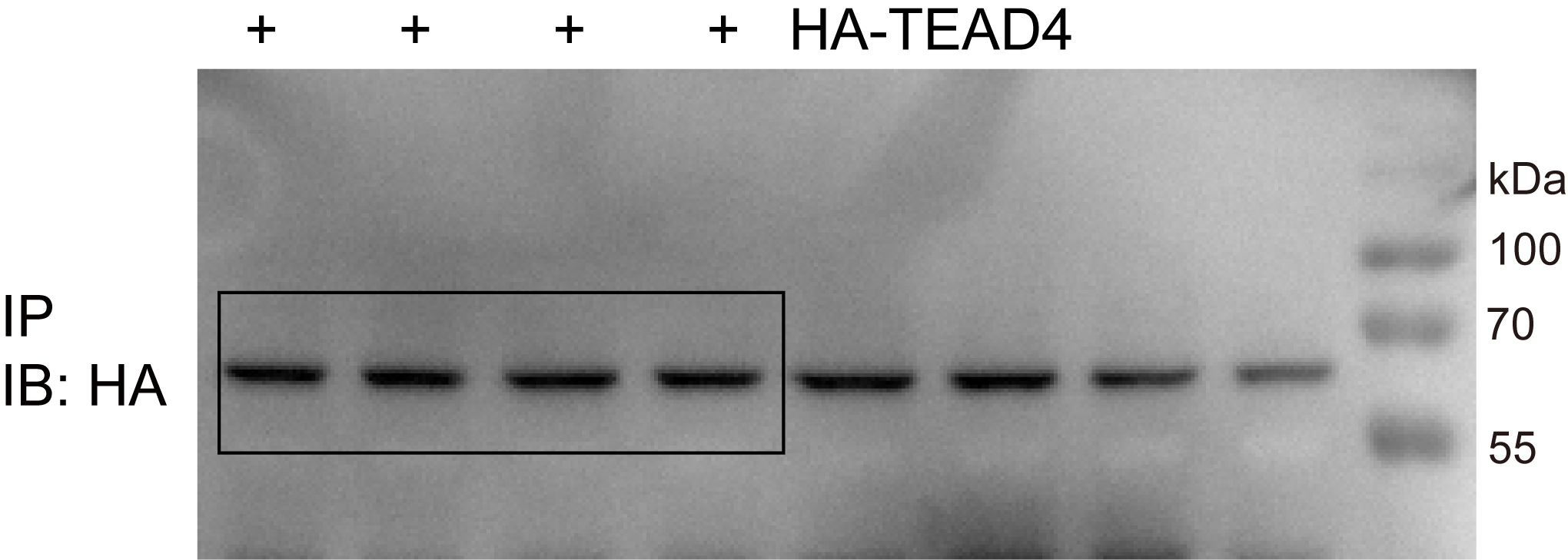

Supplement: Supplementary file 10 — Source data Fig. 3 [file 44318_2024_257_MOESM10_ESM.zip › Figure 3/3D/IP_IB_HA.tif]

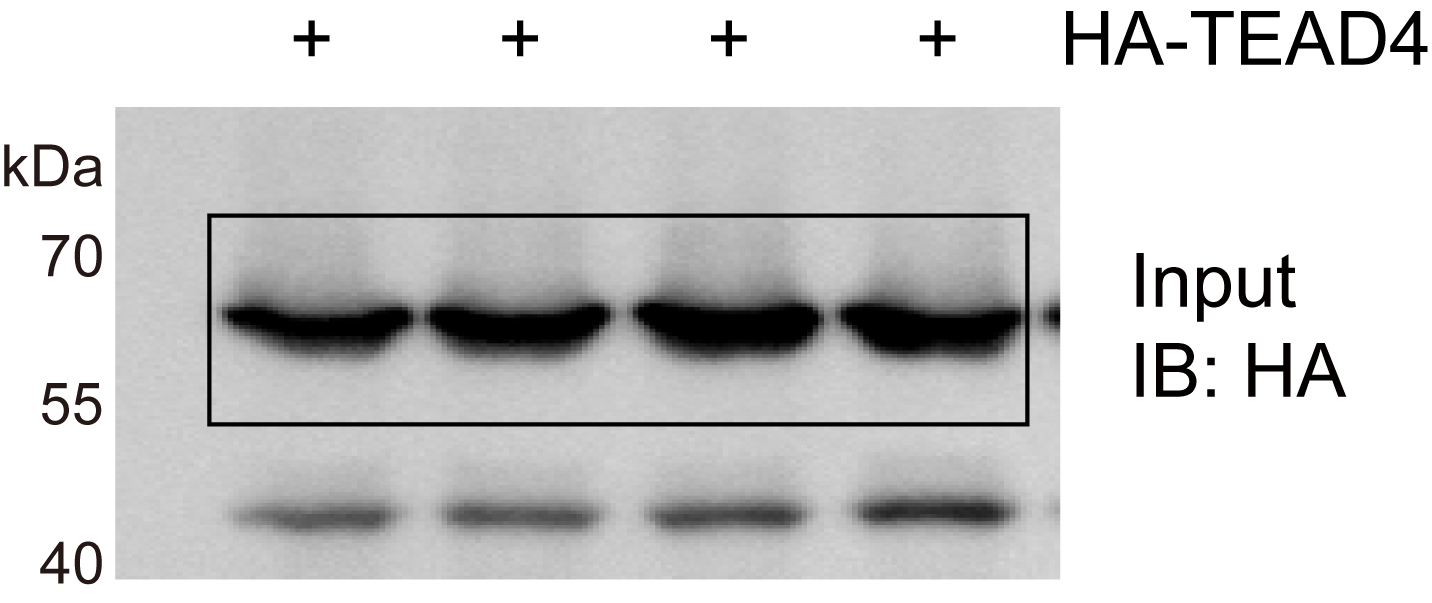

Supplement: Supplementary file 10 — Source data Fig. 3 [file 44318_2024_257_MOESM10_ESM.zip › Figure 3/3D/Input_IB_HA.tif]

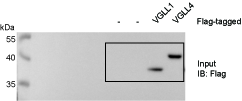

Supplement: Supplementary file 10 — Source data Fig. 3 [file 44318_2024_257_MOESM10_ESM.zip › Figure 3/3D/Input_IB_FLAG.tif]

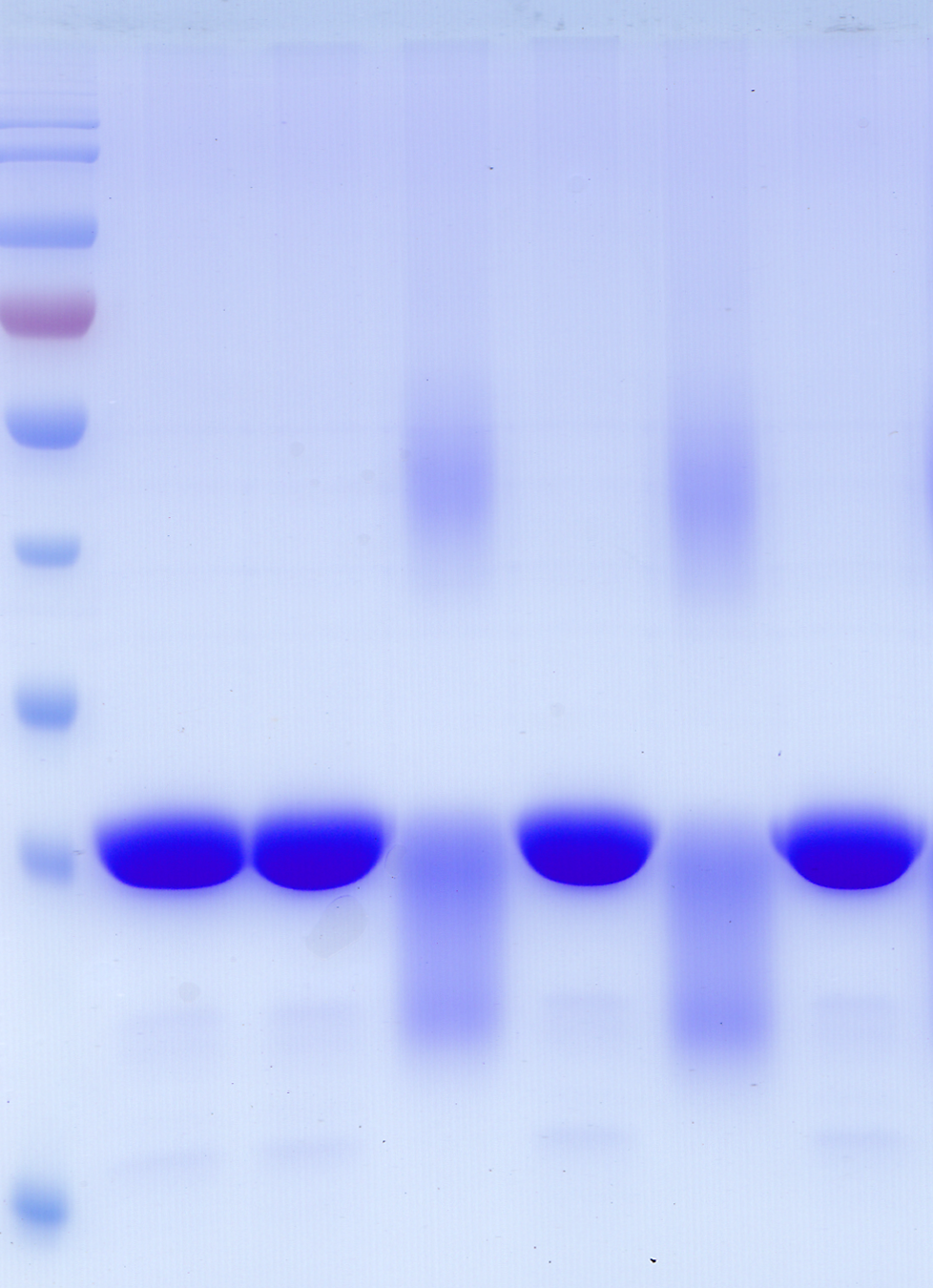

Supplement: Supplementary file 10 — Source data Fig. 3 [file 44318_2024_257_MOESM10_ESM.zip › Figure 3/3H/CBB for DSS-Crosslinking.png]

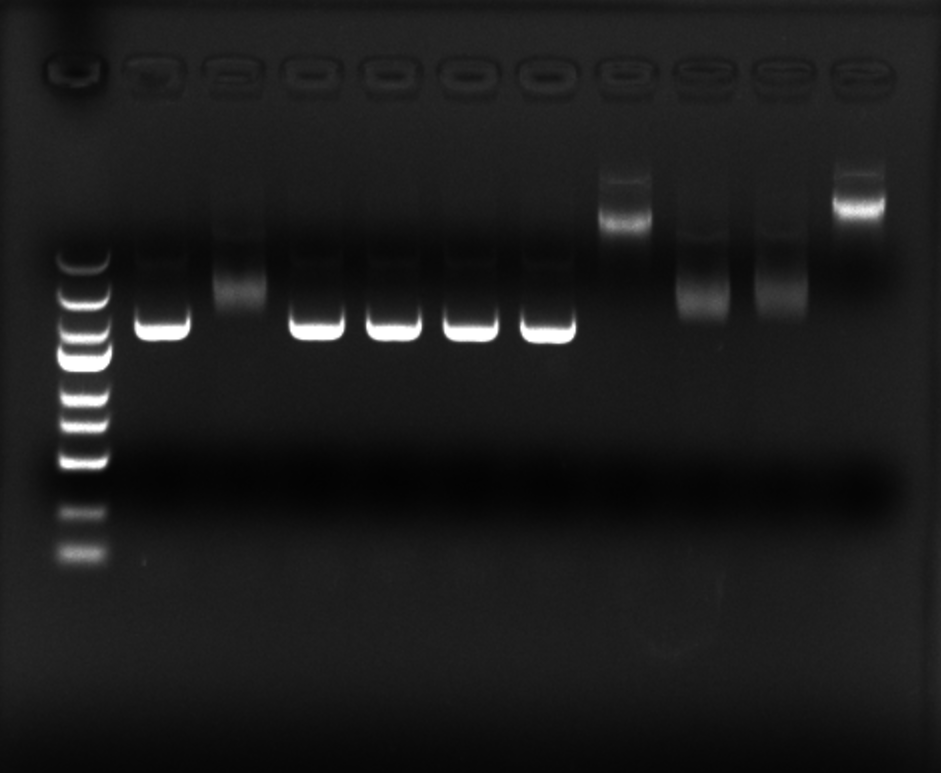

Supplement: Supplementary file 11 — Source data Fig. 4 [file 44318_2024_257_MOESM11_ESM.zip › Figure 4/4B/EMSA.tif]

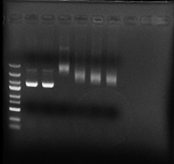

Supplement: Supplementary file 11 — Source data Fig. 4 [file 44318_2024_257_MOESM11_ESM.zip › Figure 4/4A/EMSA right.tif]

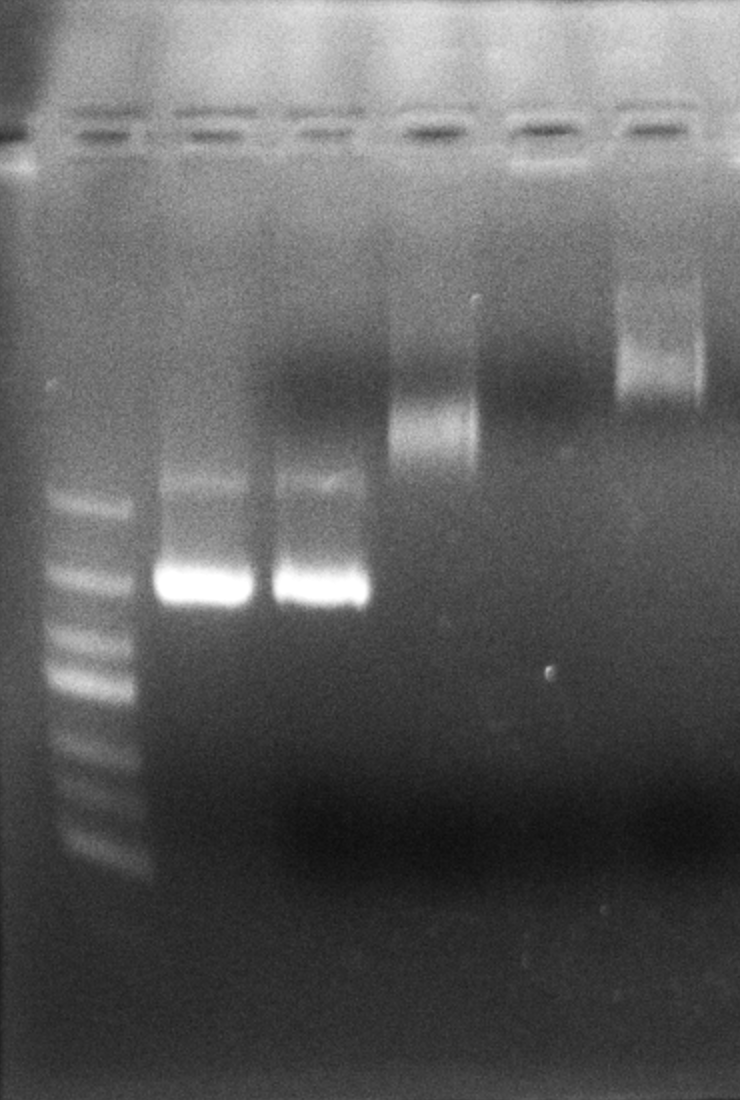

Supplement: Supplementary file 11 — Source data Fig. 4 [file 44318_2024_257_MOESM11_ESM.zip › Figure 4/4A/EMSA left.tif]

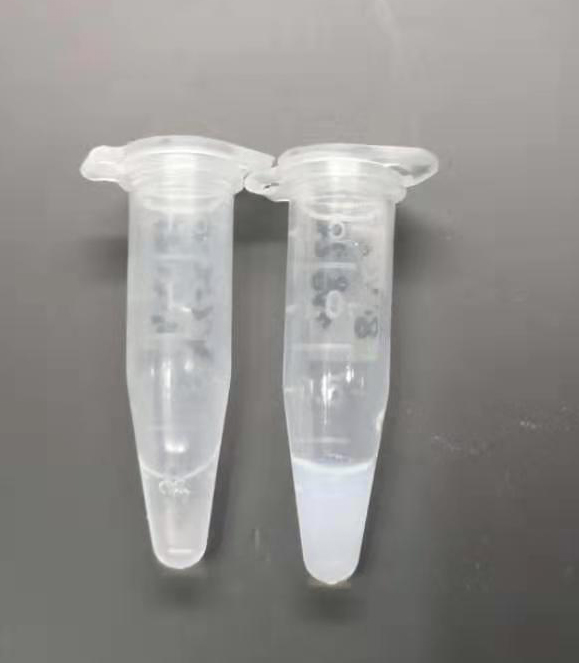

Supplement: Supplementary file 12 — Source data Fig. 5 [file 44318_2024_257_MOESM12_ESM.zip › Figure 5/5G/Photo.jpg]

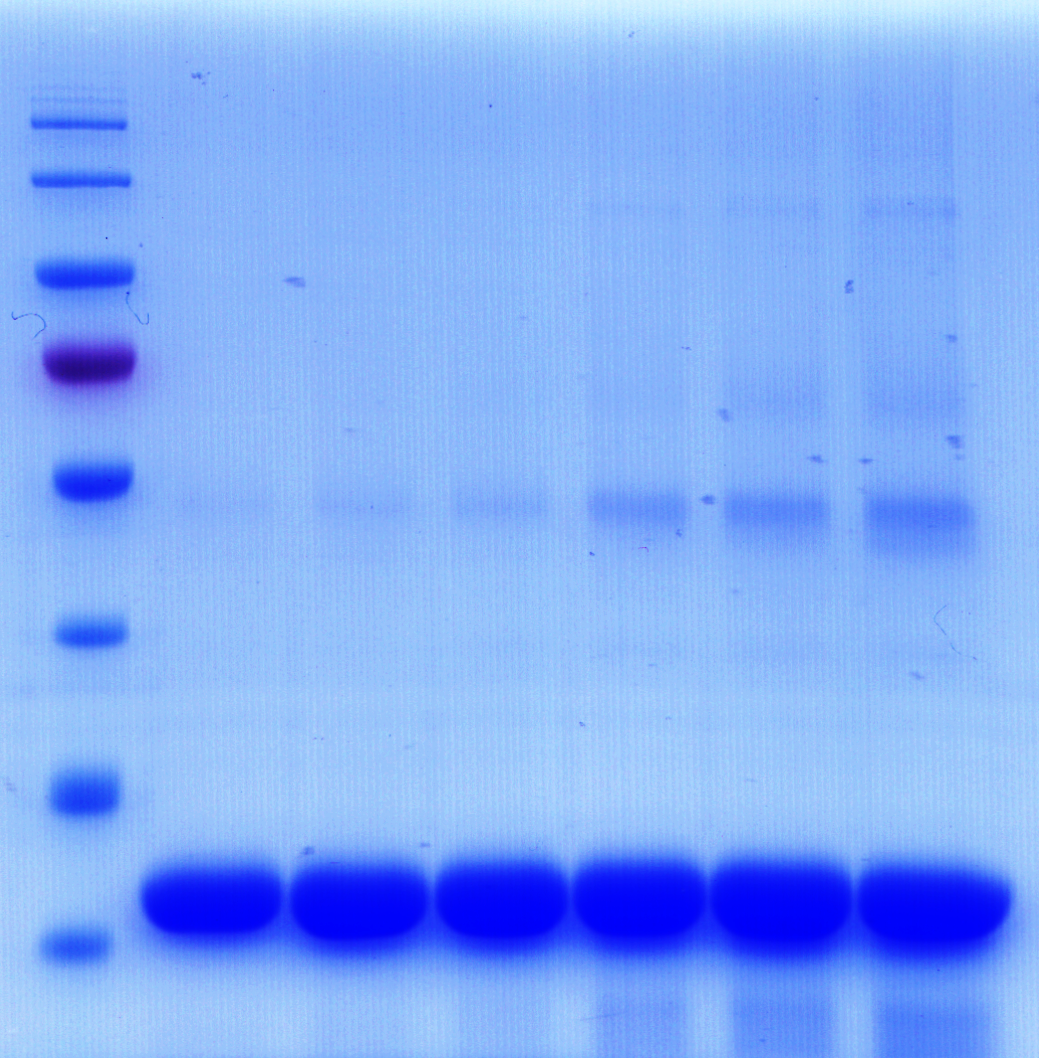

Supplement: Supplementary file 12 — Source data Fig. 5 [file 44318_2024_257_MOESM12_ESM.zip › Figure 5/5C/DSS crosslink.png]

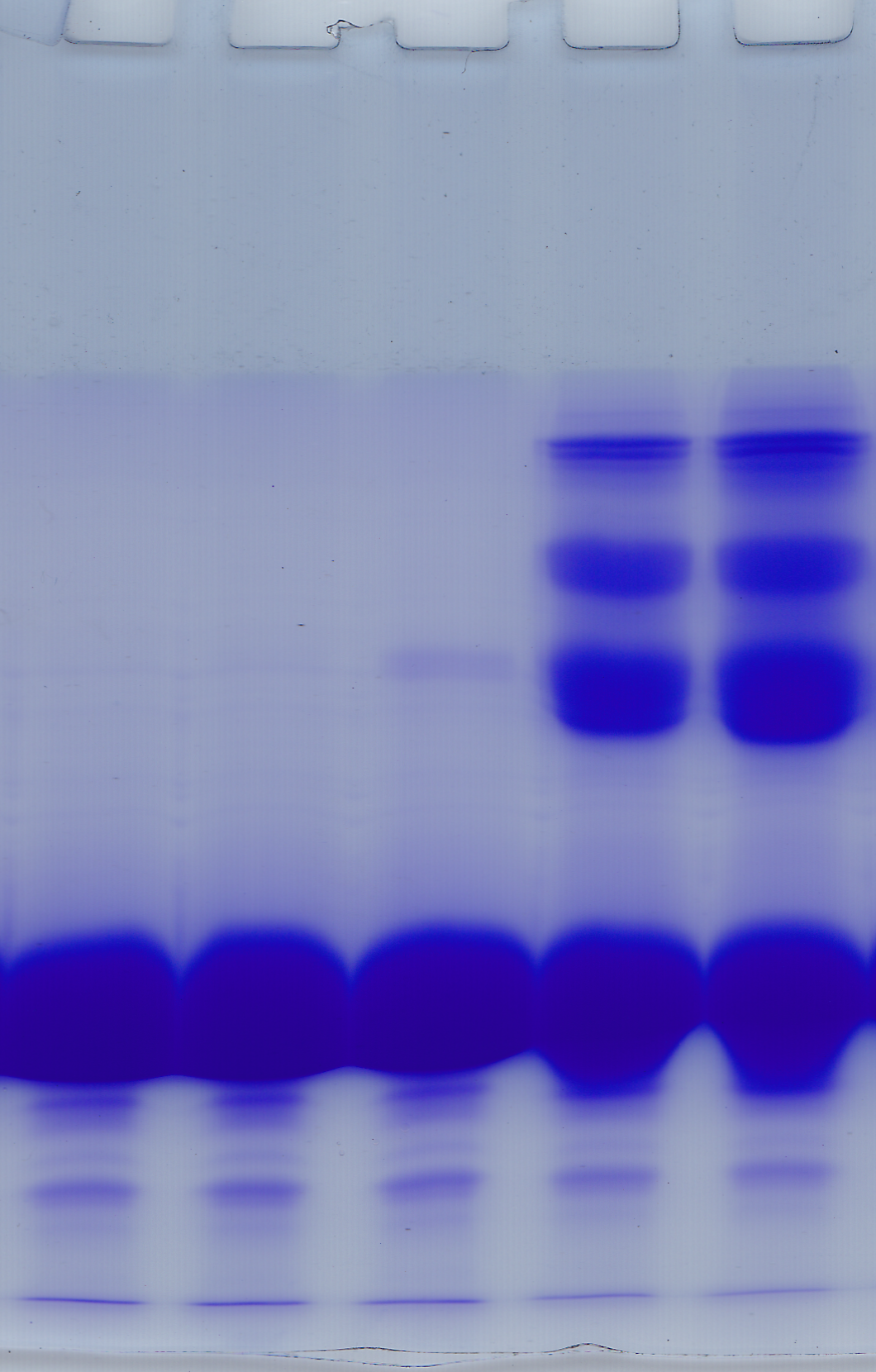

Supplement: Supplementary file 12 — Source data Fig. 5 [file 44318_2024_257_MOESM12_ESM.zip › Figure 5/5E/DSS crosslinking.png]

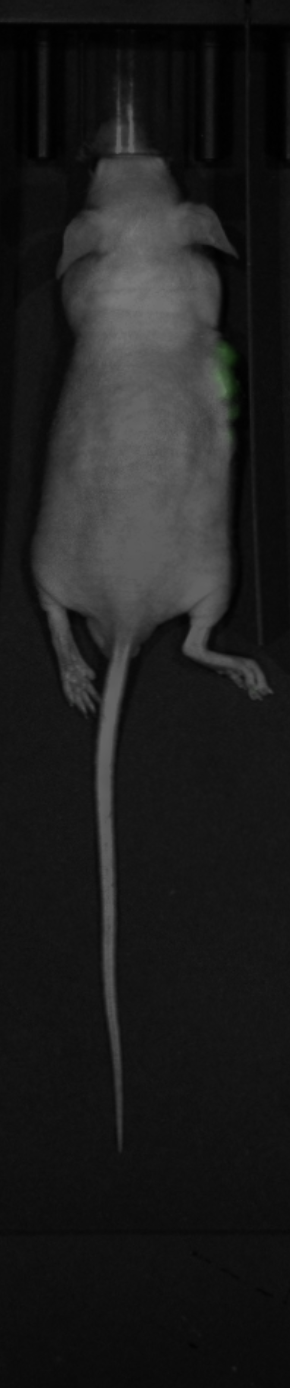

Supplement: Supplementary file 14 — Source data Fig. 7 [file 44318_2024_257_MOESM14_ESM.zip › Figure 7/7D/GLUP++.png]

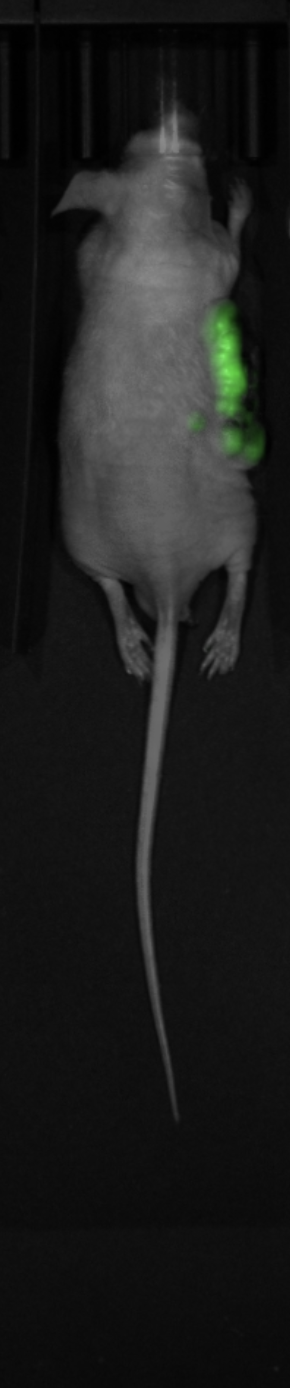

Supplement: Supplementary file 14 — Source data Fig. 7 [file 44318_2024_257_MOESM14_ESM.zip › Figure 7/7D/Ctrl.png]

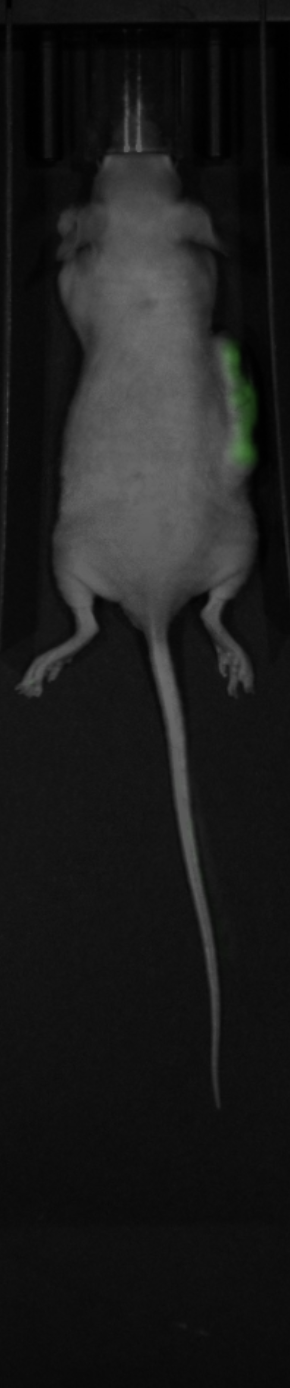

Supplement: Supplementary file 14 — Source data Fig. 7 [file 44318_2024_257_MOESM14_ESM.zip › Figure 7/7D/GLUP+.png]

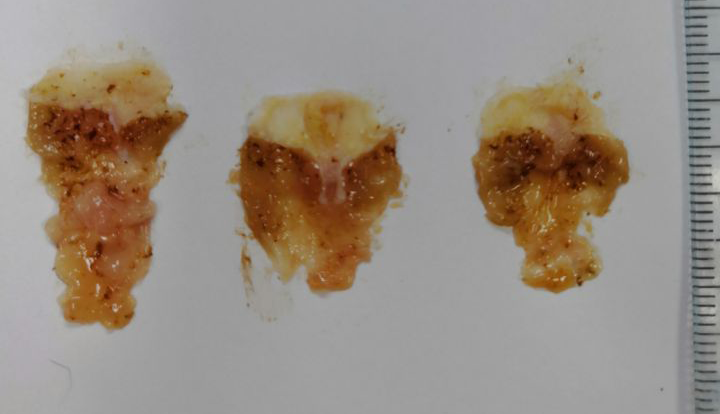

Supplement: Supplementary file 14 — Source data Fig. 7 [file 44318_2024_257_MOESM14_ESM.zip › Figure 7/7A/Ctrl-1 GLUP-5 GLUP-Lower.tif]

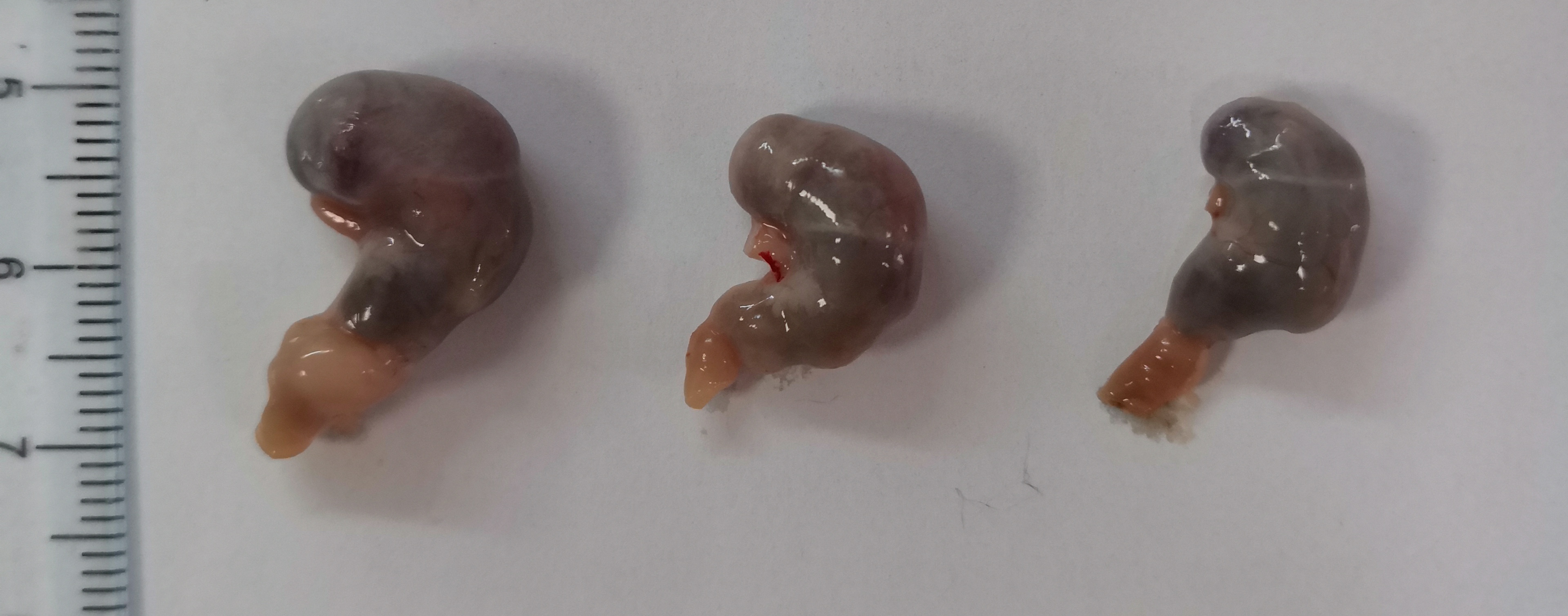

Supplement: Supplementary file 14 — Source data Fig. 7 [file 44318_2024_257_MOESM14_ESM.zip › Figure 7/7A/Ctrl-1 GLUP-5 GLUP-Upper.tif]

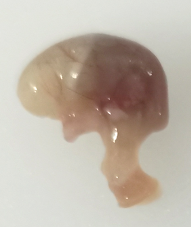

Supplement: Supplementary file 15 — Source data Fig. 8 [file 44318_2024_257_MOESM15_ESM.zip › Figure 8/8I/TEAD4-Low-GLUP.tif]

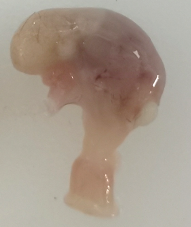

Supplement: Supplementary file 15 — Source data Fig. 8 [file 44318_2024_257_MOESM15_ESM.zip › Figure 8/8I/TEAD4-High-GLUP.tif]

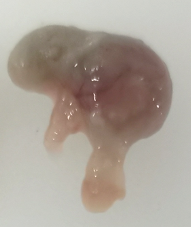

Supplement: Supplementary file 15 — Source data Fig. 8 [file 44318_2024_257_MOESM15_ESM.zip › Figure 8/8I/TEAD4-Low-Ctrl.tif]

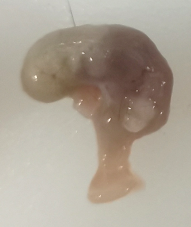

Supplement: Supplementary file 15 — Source data Fig. 8 [file 44318_2024_257_MOESM15_ESM.zip › Figure 8/8I/TEAD4-High-Ctrl.tif]

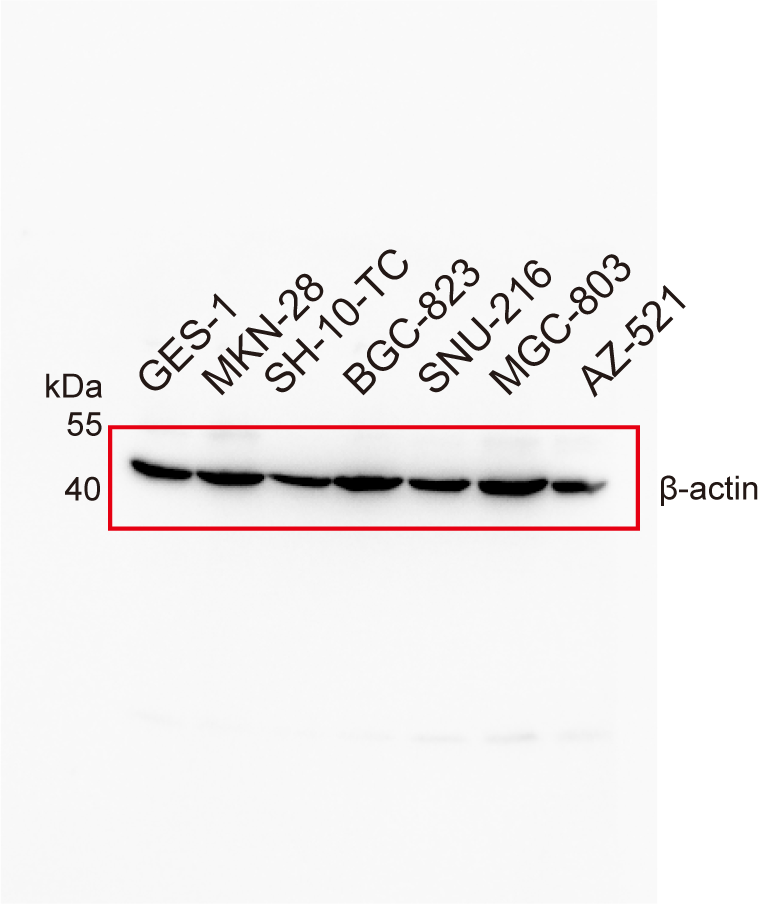

Supplement: Supplementary file 15 — Source data Fig. 8 [file 44318_2024_257_MOESM15_ESM.zip › Figure 8/8C/Actin 7 cell lines.tif]

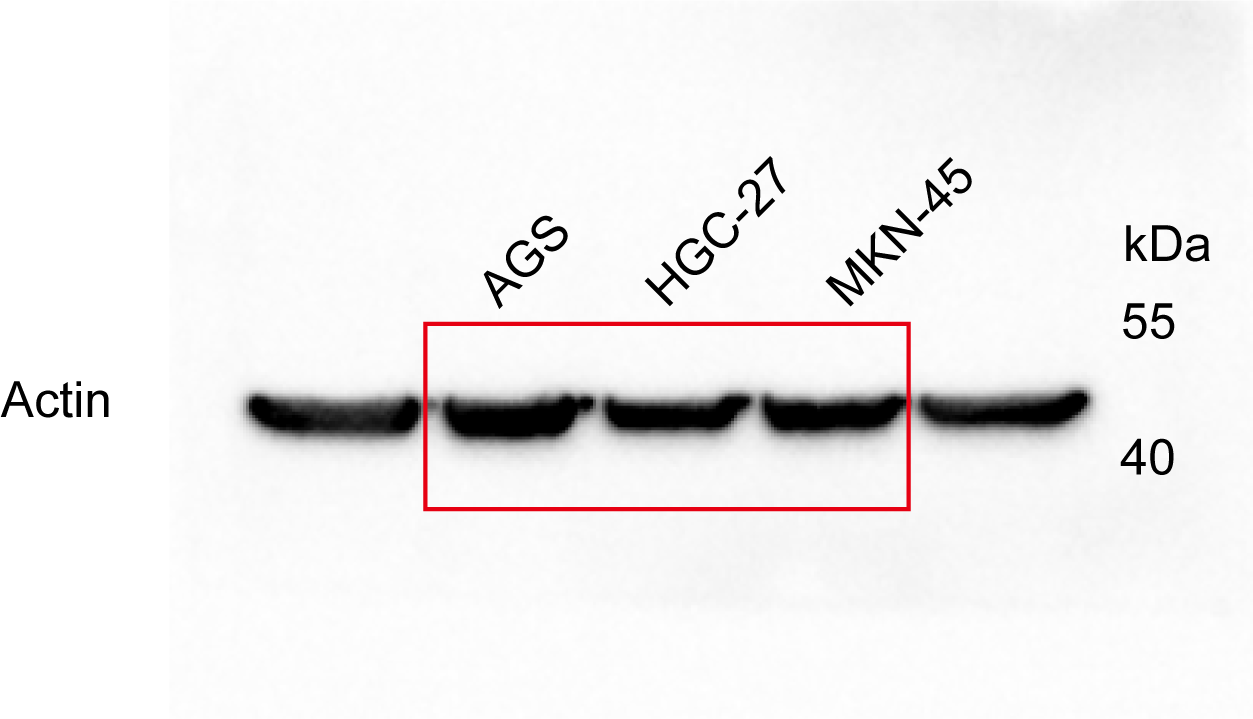

Supplement: Supplementary file 15 — Source data Fig. 8 [file 44318_2024_257_MOESM15_ESM.zip › Figure 8/8C/Actin 3 cell lines.tif]

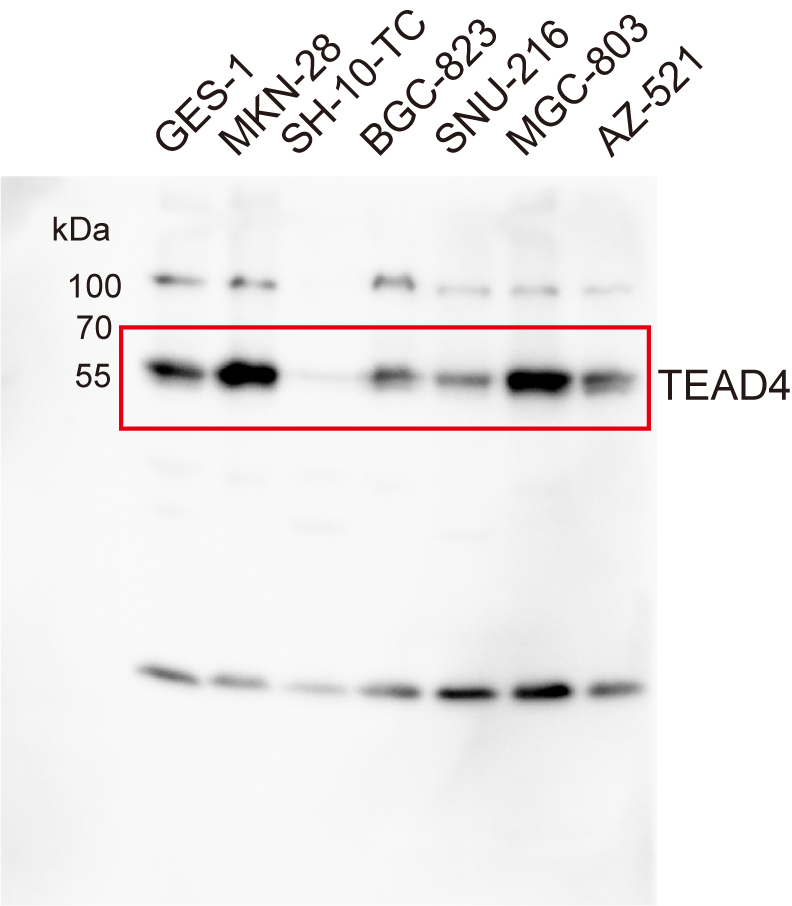

Supplement: Supplementary file 15 — Source data Fig. 8 [file 44318_2024_257_MOESM15_ESM.zip › Figure 8/8C/TEAD4 7 cell lines.tif]

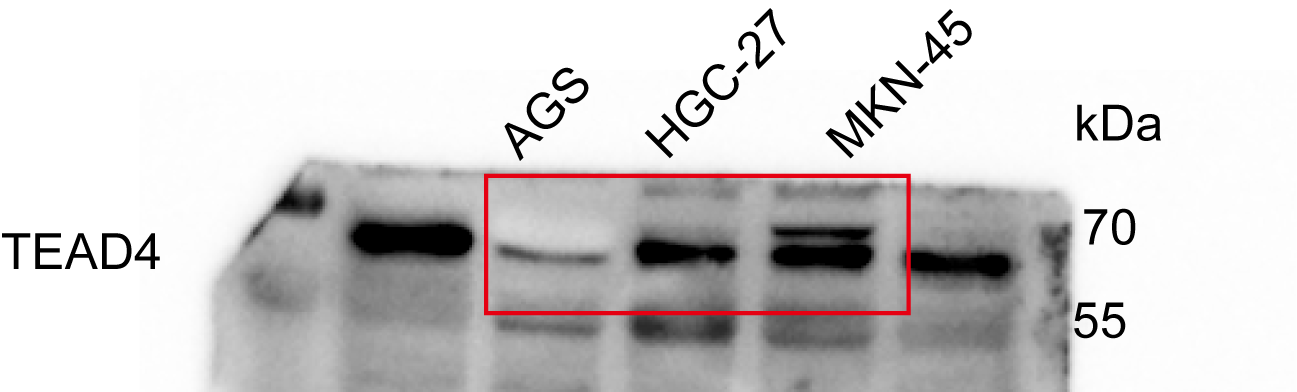

Supplement: Supplementary file 15 — Source data Fig. 8 [file 44318_2024_257_MOESM15_ESM.zip › Figure 8/8C/TEAD4 3 cell lines.tif]

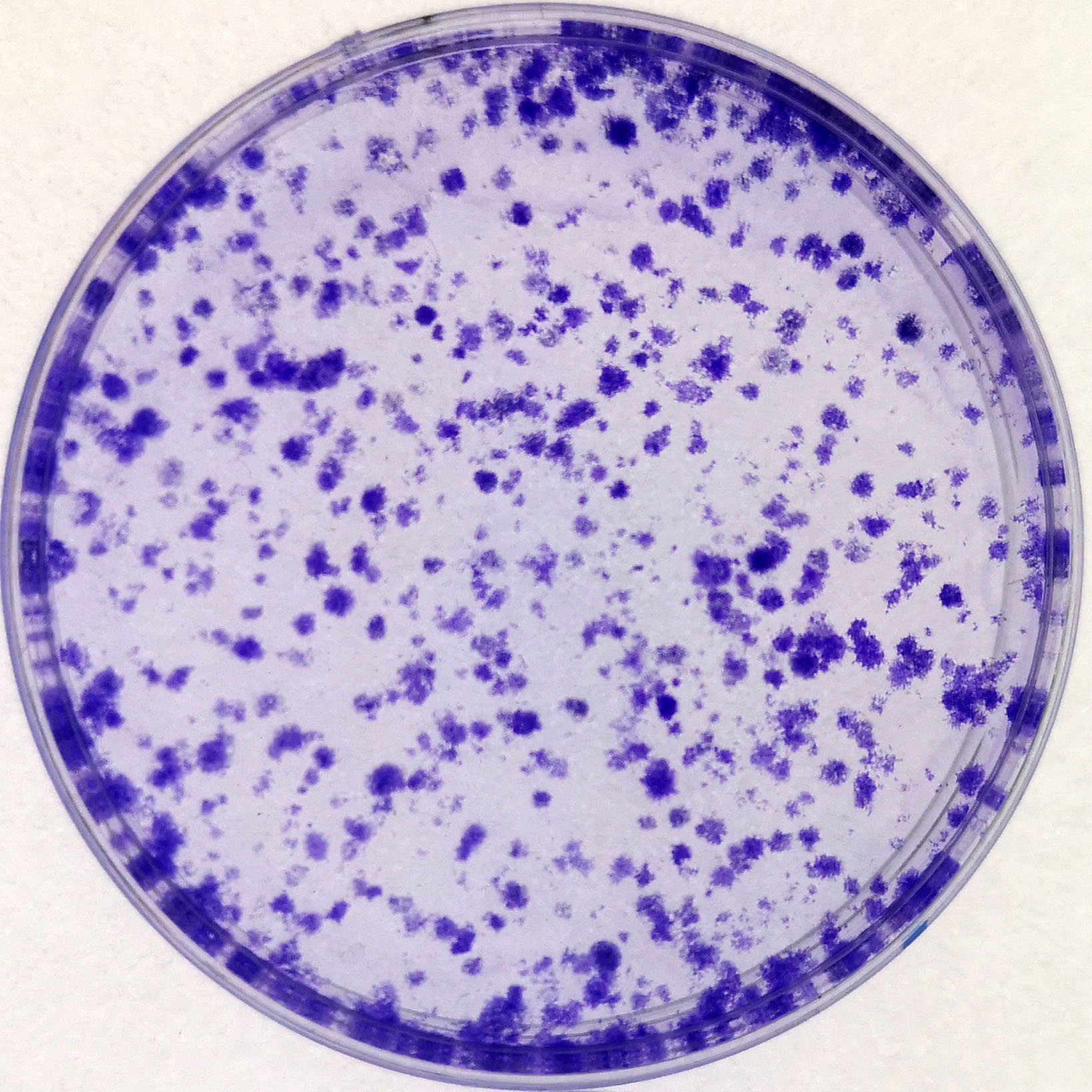

Supplement: Supplementary file 15 — Source data Fig. 8 [file 44318_2024_257_MOESM15_ESM.zip › Figure 8/8B/Ctrl.jpg]

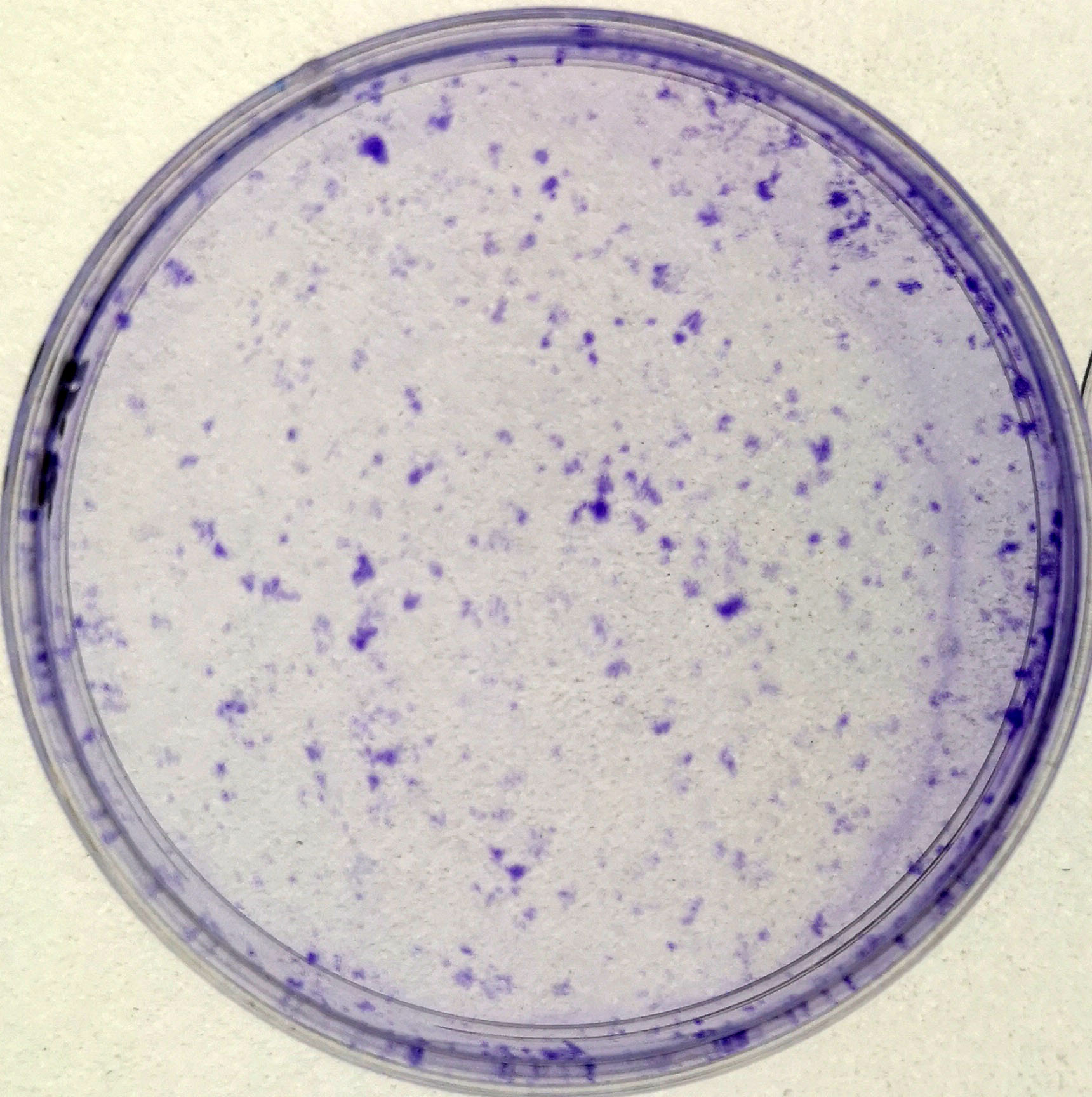

Supplement: Supplementary file 15 — Source data Fig. 8 [file 44318_2024_257_MOESM15_ESM.zip › Figure 8/8B/GLUP.jpg]
